# Association of maternal thyroid function and gestational diabetes with pregnancy outcomes: a retrospective cohort study

**DOI:** 10.3389/fendo.2025.1555409

**Published:** 2025-06-06

**Authors:** Chang Zou, Qinxin Shen, Yuanyuan Yang, Yini Liao, Qiaoling Du

**Affiliations:** Shanghai Key Laboratory of Maternal Fetal Medicine, Shanghai Institute of Maternal-Fetal Medicine and Gynecologic Oncology, Shanghai First Maternity and Infant Hospital, School of Medicine, Tongji University, Shanghai, China

**Keywords:** gestational diabetes mellitus, hypothyroidism, birth weight, hypertensive disorder, preterm birth

## Abstract

**Background:**

Gestational diabetes mellitus (GDM) and thyroid dysfunction share demographic overlap in at-risk populations, both exerting adverse effects on pregnancy. Their combined influence on pregnancy outcomes and complications requires further investigation through large-scale clinical studies.

**Objective:**

This study aimed to compare trimester-specific thyroid function in a GDM population with a euthyroid population, and to examine the impact of maternal thyroid function on pregnancy outcomes after adjusting for GDM diagnosis status.

**Methods:**

The retrospective cohort study involved singleton pregnant women registered between 2013 and 2020 in Shanghai, China. Maternal and infant biometrics were extracted from the electronic system at Shanghai First Maternity and Infant Hospital. The primary statistical methods in the study include logistic regression model and parallel mediation analyses.

**Results:**

Of the 81,488 pregnancies included, 8,868 had GDM. Compared to the population without GDM, the GDM population exhibited lower free thyroxine (FT4) and different elevated thyroid-stimulating hormone (TSH) levels in specific trimesters. Mid-pregnancy thyroid function correlated with risks of preterm birth [for FT4<2.5th percentile while TSH>97.5th, adjusted odds ratio (OR)=2.471, 95% confidence interval (CI): 1.234−4.478] and fetal overgrowth (for FT4<2.5th percentile, adjusted OR=1.551, 95% CI: 1.271–1.874). Late-pregnancy low FT4 was associated with hypertensive disorders (for FT4<2.5th percentile while TSH>97.5th, adjusted OR=3.279, 95% CI: 1.221−7.375; for isolated FT4<2.5th percentile, adjusted OR=2.010, 95% CI: 1.260−3.057). GDM amplified all these risks. Moreover, maternal ferritin was a primary mediator in thyroid-neonatal weight associations, particularly in late pregnancy (mediation proportion: 22.1%).

**Conclusion:**

This study highlighted the increased risk of adverse outcomes associated with thyroid dysfunction in GDM pregnancies, underscoring the necessity for combined thyroid function and glucose metabolism screening, which facilitates timely interventions to mitigate risks of preterm birth, hypertensive disease, and fetal overgrowth.

## 1 Introduction

As a common chronic disease during pregnancy, gestational diabetes mellitus (GDM) poses an increasing threat to the health of pregnant women worldwide. Substantial evidence demonstrates that GDM adversely impacts maternal health through inducing metabolic abnormalities (1–3) and higher risks of adverse pregnancy outcomes, including macrosomia, large for gestational age (LGA) infants (4), cesarean delivery, and preterm birth (5).

Remarkably, glucose homeostasis during pregnancy may be associated with the physiological adaptation of maternal thyroid function, though this relationship requires further investigation (6). In addition to fetal neurodevelopment, adequate maternal thyroid function is also essential for fetal growth (7), and thyroid dysfunction in pregnant women can lead to increased risk of adverse outcomes, including gestational hypertension or preeclampsia, preterm birth, and fetal growth abnormalities (8, 9) Thyroid dysfunction, especially hypothyroidism-related diseases (Hypo-RD), such as isolated maternal hypothyroxinemia (IMH), subclinical hypothyroidism, and clinical hypothyroidism, and GDM represent the most prevalent endocrinopathies in pregnancy, constituting key focus areas in obstetric endocrinology. Disordered glucose metabolism and thyroid dysfunction co-occur frequently (10, 11), due to the intricate relationship between thyroid function and glucose metabolism regulation (12, 13), and the shared risk factors include advanced conception age, overweight or obesity, and autoimmune predisposition (14).

While the increased risk of glucose metabolism disorders in pregnant women with thyroid dysfunction is well-established (15), the precise characteristics of thyroid function alterations in GDM remain incompletely characterized. There are existing studies reporting elevated thyroid-stimulating hormone (TSH) levels, higher thyroid peroxidase antibody (TPOAb) positivity rates, and decreased free thyroxine (FT4) concentrations in patients with GDM (11, 16). However, given the dynamic changes in thyroid function across gestation, each trimester exhibits distinct physiological patterns and clinical implications (17). Critically, thyroid dysfunction manifests differential clinical impacts on maternal-fetal outcomes depending on gestational timing (17), while few studies have focused on the association between second- and third-trimester disturbances and obstetric complications. These trimester-specific risk profiles necessitate validation in large cohorts with rigorous trimester stratification. The diagnostic criteria for GDM (18–20) and thyroid disorders (21, 22) keep evolving over time, and thus previous related studies are outdated and no longer align with the characteristics of the current population.

In this large-scale retrospective cohort study of Chinese pregnant women, we systematically characterized trimester-specific variations in thyroid function between GDM and non-GDM pregnancies and quantified the synergistic effects of concurrent thyroid dysfunction and GDM on adverse pregnancy outcomes. Our findings provide robust clinical evidence for understanding how glucose metabolism status modifies thyroid function-outcome relationships, offering critical insights for developing targeted screening protocols and timely therapeutic interventions in high-risk pregnancies.

## 2 Materials and methods

### 2.1 Study design, participants, and ethics approval

**Figure 1:**
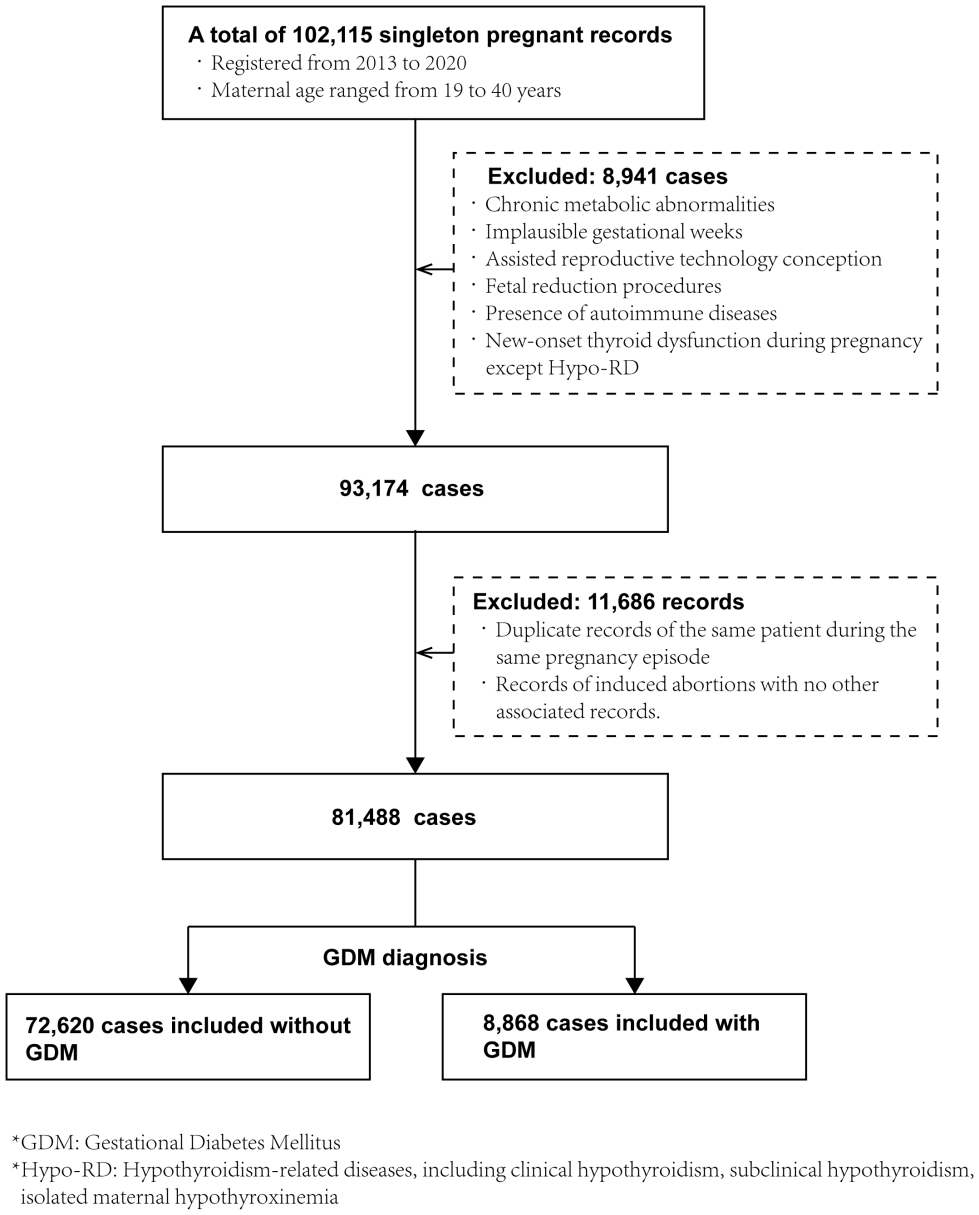
Participant recruitment and cohort formation.

This historical cohort research study was conducted at Shanghai First Maternity and Infant Hospital in China. We analyzed the electronic medical records of singleton pregnancies in women aged 19–40 years registered between January 2013 and December 2020. Exclusion criteria included: 1) Pre-existing hypertension or thyroid surgery history; 2) Other metabolic disorders (polycystic ovary syndrome, hyperlipidemia); 3) Implausible gestational age estimates; 4) Assistant reproductive technique (ART) conception or multifetal pregnancy reduction; 5) Autoimmune disease (e.g., systemic lupus erythematosus); 6) Incident maternal thyroid dysfunction during pregnancy (excluding Hypo-RD); 7) Therapeutic termination. After removing duplicate pregnancy records, 81,488 eligible cases were included (**Figure 1**). Demographic and clinical data were prospectively collected through standardized clinical documentation, including age at conception, residential origin (divided into Shanghai/non-Shanghai), parental family history of hypertension/diabetes, ethnicity (Han/non-Han), employment status (employed/unemployed), obstetric history (abortion types, preterm deliveries, parity), pre-pregnancy anthropometrics (height, weight), and calculated body mass index [BMI = weight(kg)/height(m)²]. Gestational age was determined by last menstrual period. Delivery outcomes (mode of delivery) and neonatal parameters (sex, birth weight, Apgar scores) were retrieved from electronic records. This study received approval from the Ethics Committee of the Shanghai First Maternity and Infant Hospital, School of Medicine, Tongji University (Approval Number: KS1998).

### 2.2 Diagnostic classification and outcome definitions

TPOAb positivity was defined as >60 IU/mL. According to the American Thyroid Association (ATA) guideline (22), trimester-specific FT4 and TSH reference ranges were established after excluding TPOAb-positive cases and those with thyroid disease history (**Supplementary Table S1**). Pregnancy was stratified into trimesters: first (within 13 weeks), second (13–27^+6^ weeks), and third (≥28 weeks). The diagnosis of GDM depended on the American Diabetes Association guidelines (23). GDM was diagnosed by excessive fasting plasma glucose, 1-hour glucose, or 2-hour glucose. Hypertensive disorders of pregnancy (HDP) were identified using the guidelines of the Chinese Society of Obstetrics and Gynecology during the study period (24). Data on fetal macrosomia (birth weight ≥4 kg) and LGA were extracted from discharge records.

### 2.3 Laboratory procedures

Fasting venous blood samples were centrifuged (3,000 rpm ×10 min) and stored at -80°C until analysis. Thyroid function tests (FT4, TSH, TPOAb) were performed using chemiluminescent immunoassays (ADVIA Centaur XP, Siemens). Serum ferritin was quantified with Beckman Coulter kits on UniCel DxI 800 analyzers. 25-hydroxyvitamin D levels were measured via chemiluminescent microparticle immunoassay (Architect i2000SR, Abbott). For each participant, we calculated the average values of blood tests (FT4, TSH, ferritin, 25-hydroxyvitamin D) for each trimester based on all available results within that period.

### 2.4 Covariate classification

Pre-pregnancy BMI was categorized according to the Chinese standard: underweight (<18.5 kg/m^2^), normal (≥18.5, <24 kg/m^2^), overweight/obesity (≥24 kg/m^2^). Advanced maternal age was defined as ≥35 years. Delivery modes were dichotomized as spontaneous vaginal delivery (with/without instrumentation) versus cesarean section. Missing data were excluded from the analysis.

### 2.5 Statistical analyses

Continuous variables underwent normality assessment using Kolmogorov–Smirnov tests. Non-normally distributed data were expressed as median (interquartile range), and categorical variables as counts (percentages). Group comparisons employed Mann–Whitney U tests (non-normal), Student’s t-tests (normal), χ² tests (expected frequencies ≥5), or Fisher’s exact tests. Multivariable logistic regression models were adjusted for clinically relevant confounders identified through univariate screening. Parallel mediation analyses using the SPSS PROCESS macro (Model 4) examined the ferritin/vitamin D-mediated relationships between thyroid parameters (TSH/FT4) and birth weight. Bootstrap resampling (5,000 iterations) generated 95% confidence intervals for direct/indirect effects. Mediation proportion was calculated as (indirect effect/total effect)×100%. Analyses were stratified by trimester with a significance threshold at p<0.05. All analyses were performed using IBM SPSS 26.0 (Armonk, NY).

## 3 Results

### 3.1 Basic characteristic of the study population

A total of 102,115 cases were enrolled, and ultimately, 81,488 were included to establish a historical cohort. Records with a discharge diagnosis of “gestational diabetes mellitus” were assigned to the GDM group (totaling 8,868), while the remaining records were divided into the non-GDM group (totaling 72,620) (**Figure 1**). **Table 1** presents the baseline characteristics of the participants stratified by GDM diagnosis status. As expected, the participants in the GDM group were more likely to be ≥30 years and have a family history of hypertension or diabetes. The prevalence of overweight or obesity was also significantly higher in the GDM group compared to the non-GDM group. Additionally, gestational age at delivery was shorter in the participants in the GDM group, and the rate of spontaneous vaginal delivery (without instrumental assistance) was significantly lower (57.4% vs. 63.9%). Notably, the prevalence of TPOAb positivity was similar between the two groups.

**Table 1:** Baseline characteristics.

Basic information	Overall (n=81,488)	Patients with GDM (n=8,868)	Non-GDM patients (n=72,620)
Age at conception (years)	30 (28, 32)	31 (29, 34)	30 (28, 32)
≤24	3,622 (4.4%)	158 (1.8%)	3,464 (4.8%)
25-29	34,088 (41.8%)	2,867 (32.3%)	31,221 (43.0%)
30-34	33,391 (41.0%)	3,993 (45.0%)	29,398 (40.5%)
≥35	10,387 (12.7%)	1,876 (21.2%)	8,511 (11.7%)
Pre-pregnancy BMI^*^	20.7 (19.1, 22.5)	21.7 (19.9, 23.9)	20.5 (19.1, 22.3)
<18.5 kg/m²	11,801 (15.5%)	739 (9.0%)	11,062 (16.3%)
18.5 kg/m² - <24 kg/m²	54,126 (71.1%)	5,459 (61.6%)	48,667 (67.0%)
≥24 kg/m²	10,155 (13.3%)	2,007 (22.6%)	8,148 (11.2%)
Parental family history^*^			
None	69,903 (85.8%)	7,270 (82.0%)	62,633 (86.2%)
Only hypertension disease	9,519 (11.7%)	1,203 (13.6%)	8,316 (11.5%)
Only diabetes	1,683 (2.1%)	351 (4.0%)	1,332 (1.8%)
Both diabetes and hypertension	359 (0.4%)	69 (0.8%)	290 (0.4%)
Region (Shanghai%)	25,294 (31.0%)	3,264 (36.8%)	22,030 (30.3%)
Ethnicity (Han %)	79,620 (97.7%)	8,695 (97.8%)	70,925 (97.7%)
Employment status (unemployed %)	6,350 (7.8%)	668 (7.5%)	5,682 (7.8%)
Abortion history	0 (0, 1)	0 (0, 1)	0 (0, 1)
Spontaneous Abortion	0 (0, 0)	0 (0, 0)	0 (0, 0)
Induced Abortions	0 (0, 0)	0 (0, 0)	0 (0, 0)
Parity	1 (1, 2)	1 (1, 2)	1 (1, 2)
Preterm birth history	0 (0, 0)	0 (0, 0)	0 (0, 0)
Delivery mode (Vaginal delivery%)	51,459 (63.1%)	5,090 (57.4%)	46,369 (63.9%)
Gestational weeks	39.4 (38.6, 40.1)	39.1 (38.4, 39.9)	39.4 (38.7, 40.3)
Neonatal gender (female%)	38,386 (47.1%)	4,153 (46.8%)	34,233 (47.1%)
Birth weight	3330 (3065, 3600)	3330 (3043, 3610)	3330 (3070, 3600)
Apgar score at 1 minute	10 (9, 10)	10 (9, 10)	10 (9, 10)
Apgar score at 5 minutes	10 (10, 10)	10 (10, 10)	10 (10, 10)
TPOAB (positive %)	8,243 (10.1%)	900 (10.1%)	7,343 (10.1%)

Items marked with “*” indicate that the corresponding information for some participants was missing. Pre-pregnancy BMI data for 5,406 cases were missing; parental family disease history for 24 cases was missing. BMI, body mass index; GDM, gestational diabetes mellitus; TPOAB, thyroid peroxidase antibody.

### 3.2 The GDM population exhibited distinct thyroid function and nutritional marker profiles

The prenatal examination results stratified by GDM status are presented in **Table 2**. Significant differences were observed in thyroid function markers (TSH, FT4) and nutritional indicators (ferritin, vitamin D) between the GDM and non-GDM groups across pregnancy stages. In the GDM group, TSH and FT4 levels showed significant differences during the first and second trimesters. Specifically, in the first trimester, the GDM group exhibited higher TSH levels (p = 0.006) and lower FT4 levels (p < 0.001) compared to the non-GDM group. In the second trimester, while TSH levels increased and FT4 levels decreased in both groups, the GDM group maintained lower levels of both markers (TSH: p = 0.001; FT4: p < 0.001). No significant differences in TSH or FT4 levels were observed between the groups in late pregnancy.

**Table 2:** Distribution of thyroid function indicators and incidence of hypothyroid diseases in the participants in the GDM and non-GDM groups.

Indicator	Overall (n=81,488)	Patients with GDM (n=8,868)	Non-GDM patients (n=72,620)	P-value
First trimester				
TSH (mIU/L)	1.28 (0.74,1.96)	1.31 (0.77,2.02)	1.28 (0.74,1.96)	0.006
FT4 (pmol/L)	16.64 (15.30,18.24)	16.54 (15.13,18.07)	16.65 (15.32,18.26)	<0.001
Serum vitamin D (ng/ml)	40.40 (28.90,52.70)	41.20 (29.98,52.85)	40.30 (28.80,52.70)	0.027
Ferritin (ng/ml)	47.80 (29.20,74.40)	50.90 (32.10,79.65)	47.40 (28.90,73.80)	<0.001
Second trimester				
TSH (mIU/L)	1.39 (0.84,2.12)	1.36 (0.81,2.03)	1.40 (0.84,2.13)	0.001
FT4 (pmol/L)	15.16 (13.98,16.47)	14.93 (13.77,16.25)	15.19 (14.02,16.47)	<0.001
Serum vitamin D (ng/ml)	43.90 (31.90,57.10)	44.69 (33.10,57.60)	43.80 (31.80,57.00)	0.001
Ferritin (ng/ml)	38.10 (22.90,61.80)	40.60 (24.75,65.80)	37.70 (22.70,61.38)	<0.001
Third trimester				
TSH (mIU/L)	1.82 (1.21,2.54)	1.82 (1.24,2.52)	1.82 (1.21,2.54)	0.941
FT4 (pmol/L)	13.83 (12.69,15.14)	13.71 (12.52,15.08)	13.85 (12.72,15.15)	0.081
Serum vitamin D (ng/ml)	49.62 (34.70,67.80)	53.50 (38.02,71.29)	49.20 (34.40,67.40)	<0.001
Ferritin (ng/ml)	9.60 (6.70,15.70)	10.50 (7.10,17.28)	9.50 (6.70,15.60)	<0.001

FT4, free thyroxine; GDM, gestational diabetes mellitus; TSH, thyroid-stimulating hormone.

Additionally, maternal serum vitamin D levels progressively increased throughout pregnancy, whereas ferritin levels declined. Notably, the patients with GDM demonstrated consistently higher vitamin D and ferritin levels compared to the non-GDM patients across all gestational stages (**Table 2**).

### 3.3 Trimester-specific analysis of maternal thyroid dysfunction and pregnancy outcomes

**Figure 2:**
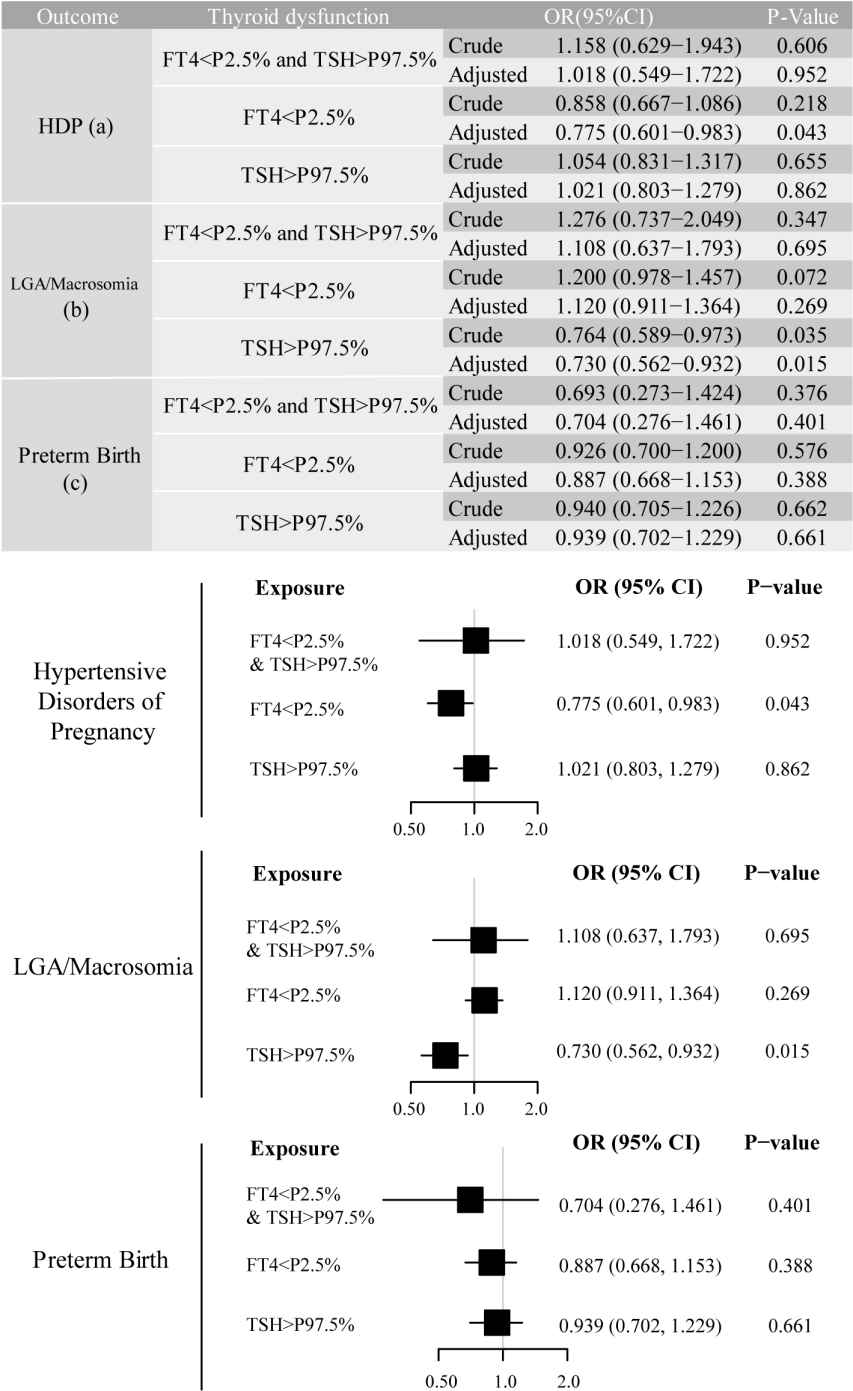
Adjusted odds ratios (95% CI) for pregnancy outcomes by first-trimester maternal thyroid function. The results were derived from logistic regression with outcome-specific covariate adjustments: hypertensive disorders of pregnancy (adjusted for maternal age, pre-pregnancy BMI, and delivery mode); large for gestational age/macrosomia (adjusted for newborn sex, pre-pregnancy BMI, and delivery mode), and preterm birth (adjusted for maternal age, newborn sex, pre-pregnancy BMI, and delivery mode), and the adjusted ORs are specifically depicted in forest plots. The regression analysis included three exposure groups divided by first-trimester FT4 and TSH levels: (1) FT4 < 2.5th percentile (P2.5); (2) TSH > 97.5th percentile (P97.5); (3) concurrent FT4 < P2.5 and TSH > P97.5. The reference group consisted of euthyroid pregnancies in this trimester. Statistical significance was set at P-value ≤ 0.01. CI, confidence interval; HDP, hypertensive disorders of pregnancy; LGA, large for gestational age; OR, odds ratio.

**Figure 3:**
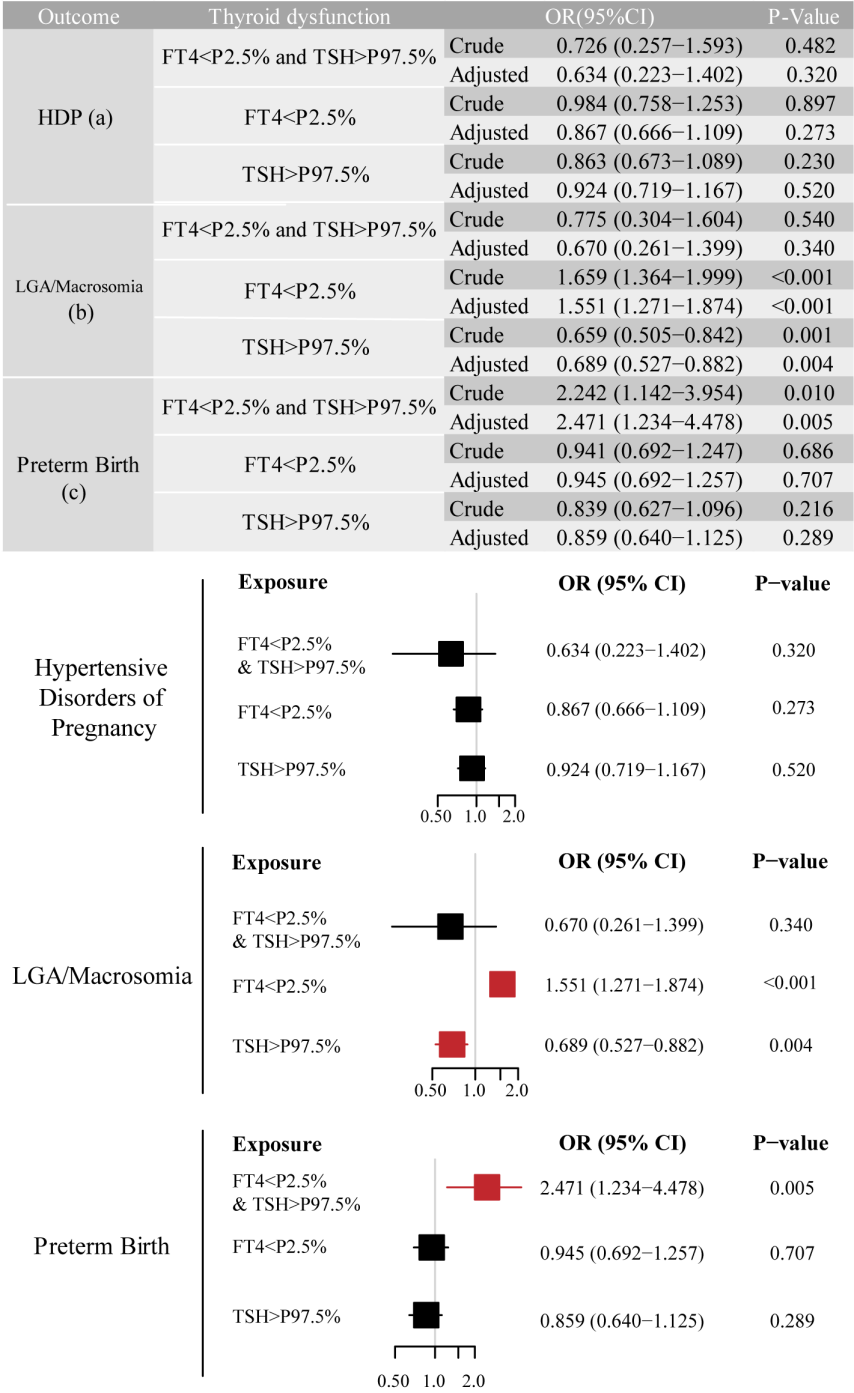
Adjusted odds ratios (95% CI) for pregnancy outcomes by second-trimester maternal thyroid function. The results were derived from logistic regression with outcome-specific covariate adjustments, as aforementioned. The adjusted ORs are specifically depicted in forest plots. The regression analysis included three exposure groups divided by second-trimester FT4 and TSH levels: (1) FT4 < P2.5; (2) TSH > P97.5; (3) concurrent FT4 < P2.5 and TSH > P97.5. The reference group consisted of euthyroid pregnancies in this trimester. Significant associations (P-value ≤ 0.01) are shown in red in the forest plots. CI, confidence interval; HDP, hypertensive disorders of pregnancy; LGA, large for gestational age; OR, odds ratio.

**Figure 4:**
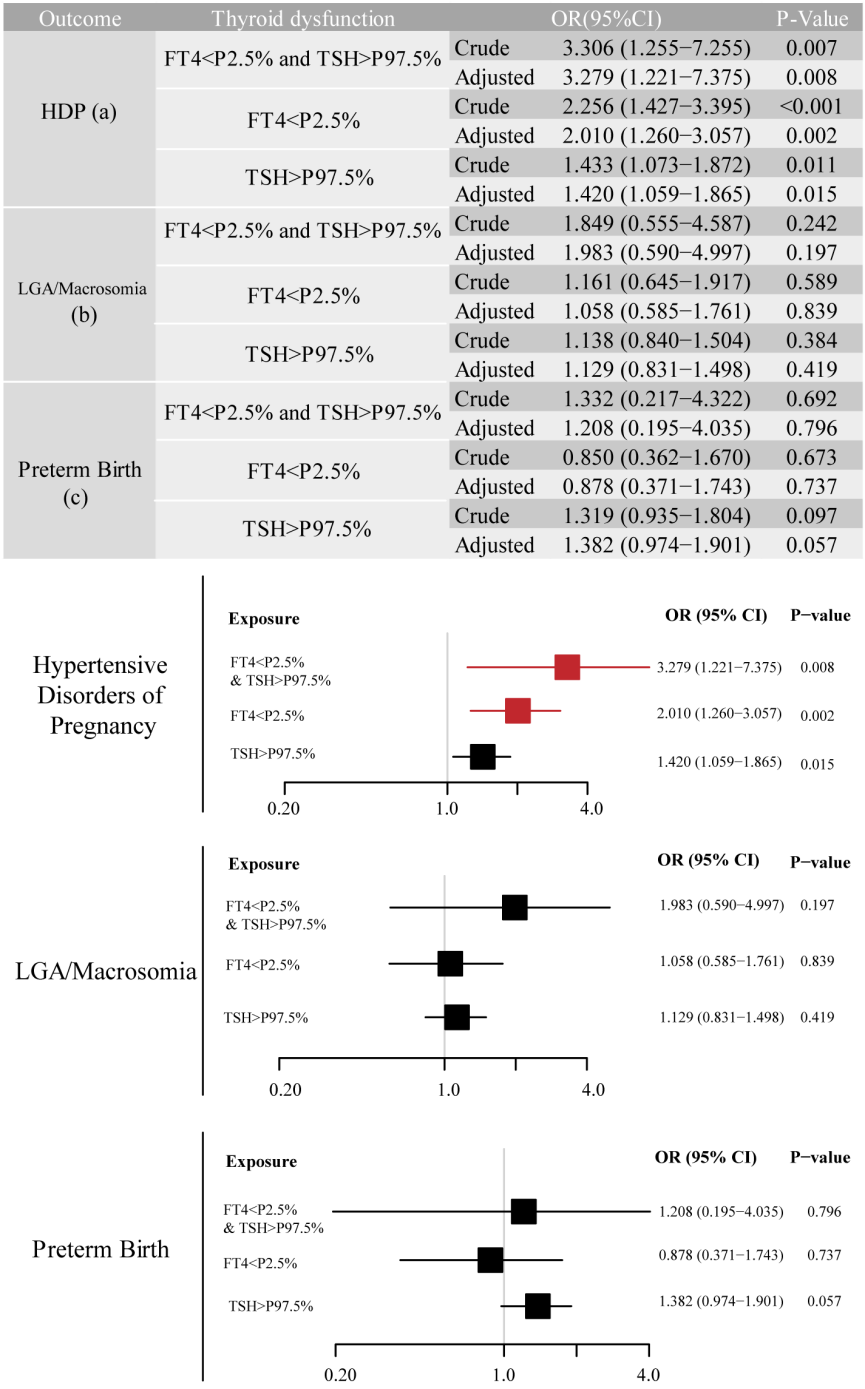
Adjusted odds ratios (95% CI) for pregnancy outcomes by third-trimester maternal thyroid function. The results were derived from logistic regression with outcome-specific covariate adjustments, as aforementioned. The adjusted ORs are specifically depicted in forest plots. The regression analysis included three exposure groups divided by third-trimester FT4 and TSH levels: (1) FT4 < P2.5; (2) TSH > P97.5; (3) concurrent FT4 < P2.5 and TSH > P97.5. The reference group consisted of euthyroid pregnancies in this trimester. Significant associations (P-value ≤ 0.01) are shown in red in the forest plots. CI, confidence interval; HDP, hypertensive disorders of pregnancy; LGA, large for gestational age; OR, odds ratio.

We identified adverse pregnancy outcomes that were significantly associated with maternal thyroid dysfunction during specific gestational trimesters, setting p<0.01 as the significance level. The primary exposure of thyroid dysfunction was classified according to trimester-specific reference ranges (**Supplementary Table S1**) into three categories: (1) isolated hypothyroxinemia (FT4<2.5th percentile [P2.5]), (2) isolated elevated TSH [>97.5th percentile (P97.5)], and (3) combined dysfunction (FT4<P2.5 and TSH>P97.5), as illustrated in the forms of a table and forest plot in **Figures 2**-**4**. The association of the three types of thyroid dysfunction with each outcome exhibited distinct trimester-specific patterns. During the first trimester, no significant relation was found between TSH, FT4, and adverse outcomes (**Figure 2**). The second-trimester analysis revealed that isolated hypothyroxinemia increased LGA/macrosomia risk by 55.1% [adjusted odds ratio (OR)=1.551, 95% confidence interval (CI): 1.271–1.874], while isolated TSH elevation showed the opposite effect (adjusted OR=0.689, 95% CI: 0.527–0.882). Notably, combined thyroid dysfunction in the second trimester demonstrated a strong association with preterm birth (adjusted OR=2.471, 95%CI: 1.234–4.478), after adjusting for confounders (**Figure 3**). The third-trimester analysis revealed particularly strong associations between thyroid dysfunction and HDP. The highest risk was observed in women with combined hypothyroxinemia and elevated TSH (FT4<P2.5% and TSH>P97.5%), demonstrating a 3.3-fold increased risk (adjusted OR=3.279, 95% CI: 1.221–7.375). Isolated hypothyroxinemia was associated with a 2.0-fold increased HDP risk (adjusted OR=2.010, 95% CI:1.260–3.057). Though it was not significant, isolated TSH elevation still showed a 1.4-fold risk increase (adjusted OR=1.420, 95% CI:1.059–1.865, p=0.015). All associations remained statistically significant after adjustment for potential confounders. The results demonstrated a robust trimester-specific relationship between thyroid dysfunction and pregnancy outcomes.

### 3.4 Comprehensive analysis of the impact of GDM and thyroid dysfunction on pregnancy outcomes

Prior to the comprehensive analyses, we also analyzed the relationship between isolated GDM status and adverse pregnancy outcomes (**Supplementary Table S2**). GDM was associated with elevated risks of HDP (adjusted OR=1.227, 95% CI:1.116–1.349) and preterm birth (adjusted OR=1.141, 95% CI:1.013–1.286). Before confounder adjustment, the OR for LGA/macrosomia in the patients with GDM was 1.223 (1.113, 1.343) compared to the non-GDM cohort, while the OR was insignificant after adjustment (adjusted OR=0.997, 95% CI: 0.905–1.098).

Based on the study cohort, we revealed that mid- and late-pregnancy thyroid function parameters demonstrated stronger associations with adverse perinatal outcomes compared to first-trimester measures. Thus, logistic regression models to evaluate the comprehensive effects of second/third-trimester maternal thyroid function, GDM, and other confounders on pregnancy outcomes were constructed. Across all the outcomes and complications, Model 1 assessed thyroid function markers alone (TSH and FT4 distribution, serving as continuous or categorical variables), Model 2 incorporated GDM status, and Model 3 included full adjustment for confounders.

**Figure 5:**
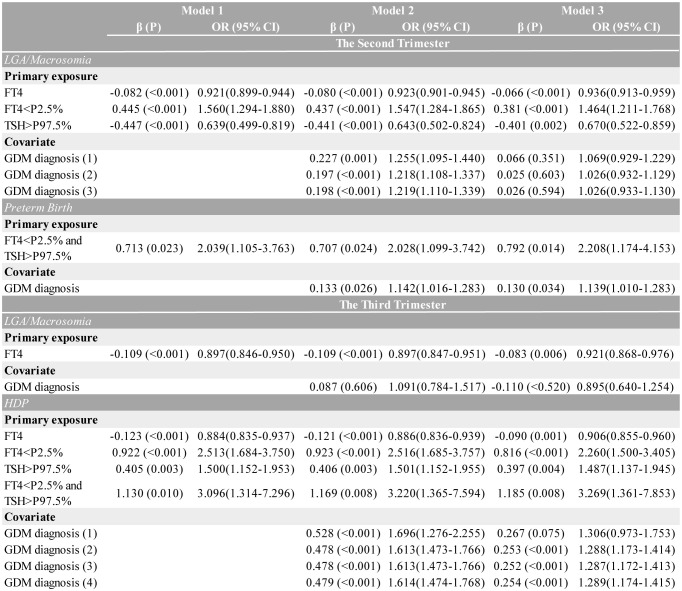
Multivariate logistic regression models presenting the association between second/third-trimester thyroid function and different outcomes, adjusted for GDM diagnosis. Three multivariable models were constructed: Model 1 examined thyroid function (using FT4 as a continuous variable; isolated low FT4, isolated high TSH, and combined low FT4/high TSH as categorical variables) alone. Model 2 added gestational diabetes (GDM) diagnosis; the superscripts (1)–(4) stand for distinct adjustments applied sequentially to each thyroid variable (listed top-to-bottom in the sub-table of each disease). Model 3 was fully adjusted for outcome-specific perinatal factors, including hypertensive disorders of pregnancy (adjusted for maternal age, pre-pregnancy BMI, and delivery mode), large for gestational age/macrosomia (adjusted for newborn sex, pre-pregnancy BMI, and delivery mode), preterm birth (adjusted for maternal age, newborn sex, pre-pregnancy BMI, and delivery mode), thyroid function, and GDM diagnosis. Only GDM ORs are displayed for covariates. CI, confidence interval; FT4, free thyroxine; OR, odds ratio.

According to **Figure 5**, continuous FT4 levels were inversely associated with LGA/macrosomia risk in all the models (fully-adjusted OR=0.936 per unit increase, 95% CI: 0.913–0.959, p<0.001). Isolated hypothyroxinemia (FT4<P2.5%) demonstrated progressively attenuated but persistently significant effects across models: crude OR=1.560 (95% CI: 1.294–1.880, p<0.001), GDM-adjusted OR=1.547 (95% CI: 1.284–1.865, p<0.001), and fully-adjusted OR=1.464 (95% CI: 1.211–1.768, p<0.001), while isolated TSH elevation showed reversed effects (OR=0.639, 95% CI: 0.499–0.819). In mid-pregnancy, for all forms of thyroid function, GDM diagnosis initially appeared to be a significant covariate, but its effect became non-significant after full adjustment. Moreover, second-trimester combined dysfunction (FT4<P2.5% and TSH>P97.5%) was associated with a 2.039-fold increased preterm birth risk (95% CI: 1.105–3.763, p=0.023), remaining significant in the fully-adjusted model (Model 3 OR=2.208, 95% CI: 1.174–4.153, p=0.014). A GDM diagnosis further increased preterm birth risk by 13.9% (95% CI: 1.010–1.283, p=0.034).

The third-trimester analyses revealed significant associations between thyroid dysfunction and HDP risk across multiple modeling approaches. As a continuous variable, each unit increase in FT4 was associated with a 9.4% reduction in HDP risk (fully-adjusted OR=0.906, 95% CI: 0.855–0.960, p=0.001). When analyzed categorically, isolated hypothyroxinemia showed progressively attenuated but persistently significant effects: crude OR=2.513 (95% CI: 1.684–3.750, p<0.001), GDM-adjusted OR=2.516 (95% CI: 1.685–3.757, p<0.001), and fully-adjusted OR=2.260 (95% CI: 1.500–3.405, p<0.001). Similarly, isolated TSH elevation maintained significant associations throughout model adjustments (crude OR=1.500, 95% CI: 1.152–1.953; fully-adjusted OR=1.487, 95% CI: 1.137–1.945). The strongest association was observed for combined dysfunction, with risk estimates increasing from the crude OR=3.096 (95% CI: 1.314–7.296) to the fully-adjusted OR=3.269 (95% CI: 1.361–7.853). While GDM diagnosis showed initial strong associations (Model 2 OR=1.613, 95% CI: 1.473–1.766), these effects were attenuated but remained significant after full adjustment (OR=1.287, 95% CI: 1.172–1.413). Notably, the predictive value of continuous FT4 levels remained stable regardless of GDM adjustment.

### 3.5 Ferritin potentially mediates thyroid-birth weight association

According to the prior result, we found a relationship between second-trimester thyroid function and LGA/macrosomia risk (as illustrated in **Figures 3** and **5**). Given the observed elevation of both vitamin D and ferritin levels in patients with GDM (**Table 2**), we performed parallel mediation analyses to examine their potential mediating roles in the thyroid function-birth weight relationship.

**Figure 6:**
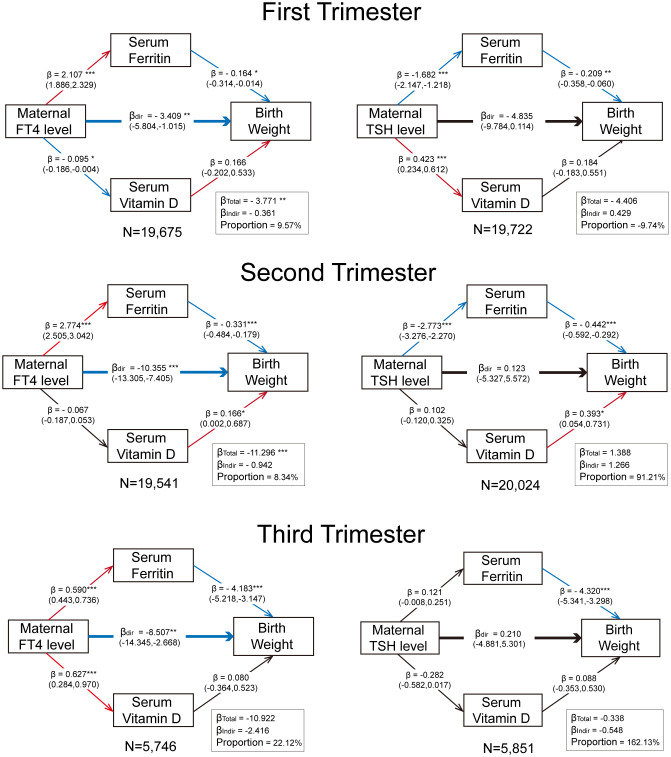
Trimester-specific mediation effect of maternal ferritin and vitamin D levels on the relationship between maternal thyroid function and birthweight. All models were adjusted for pre-pregnancy BMI and age at conception. The values in parentheses represent 95% CIs. The significance levels are indicated as follows: “*” for p < 0.05, “**” for p < 0.01, and “***” for p < 0.001. The proportion refers to the ratio of the indirect effect to the total effect. Red arrows denote significant positive correlations (p < 0.05), blue arrows denote significant negative correlations, and black arrows indicate non-significant relationships. The sample size for each analysis is displayed following “N =“.

**Figure 6** demonstrates the consistent negative correlations of maternal serum ferritin levels with neonatal birth weight across all gestational stages, and it shows the positive association with FT4 levels and negative association with TSH levels throughout pregnancy. This finding provides a potential mechanism explaining the inconsistent relationship between maternal TSH level and neonatal birthweight, especially the reduced macrosomia risk associated with an increased second-trimester isolated TSH level. The mediating effect of ferritin was most pronounced during the third trimester, where it mediated 22.12% of the total effect of third-trimester FT4 on birth weight, and the third trimester is also a critical period with the most fetal weight gain. However, no significant evidence supported vitamin D as an effective mediating factor in the relationship between maternal thyroid function and neonatal birth weight during any stage of pregnancy.

## 4 Discussion

The role of thyroid hormones in glucose metabolism is well-established, with demonstrated effects on hepatic glucose metabolism, insulin secretion, and β-cell function. These mechanisms link thyroid function to hyperglycemia and insulin resistance, even in euthyroid individuals (25–28). While clinical studies frequently associate GDM with thyroid disorders, particularly autoimmune thyroid diseases (29, 30), the underlying mechanisms remain poorly understood beyond known comorbid factors like obesity. Intriguingly, in this cohort, TPOAb positivity rates were comparable between the GDM and non-GDM groups, yet thyroid function differences persisted. This discrepancy may reflect the necessity of removing cases with pre-existing type 1 or type 2 diabetes, which helped to exclude the confounding effect of heightened autoimmunity.

Emerging evidence implicates thyroid dysfunction in increased macrosomia/LGA risk, especially hypothyroidism-related conditions (17, 31, 32). LGA fetuses frequently exhibit metabolic disturbances, the reduced placental transfer of thyroid hormones triggers compensatory fetal hypothalamic-pituitary-thyroid axis activation and increased endogenous fetal thyroid hormone production. This results in accelerated fetal metabolism with enhanced lipolysis and altered glucose utilization, promoting excessive growth (33). Concurrently, elevated fetal leptin levels and insulin-like growth factor (IGF) axis dysregulation further contribute to macrosomia (34). The metabolic profile includes increased oxidative stress and pro-inflammatory cytokine activation, which may impair placental vascular function and nutrient transport efficiency (35). These metabolic adaptations predispose to neonatal hypoglycemia and altered adipokine profiles, with potential long-term implications for obesity risk and metabolic dysfunction in offspring (36, 37). However, our findings suggest that assessing fetal growth solely by neonatal birth weight may lead to the neglect of metabolic issues in offspring of pregnant women with concurrent GDM and thyroid dysfunction, especially in those with normal pre-pregnancy weight.

After adjusting for confounders (such as pre-pregnancy BMI), the difference in LGA/macrosomia rates among the women with GDM and the non-GDM women became statistically insignificant, indicating that it was probably caused by the oversupply of maternal fatty acids and amino acids due to higher BMI, or the genetic and gender characteristics of the infant, more than GDM itself (38). Meanwhile, it was well-established in our study that the GDM cohort exhibited elevated maternal serum ferritin, while high level of ferritin was simultaneously accompanied by high FT4 and low TSH levels. As for ferritin, it exhibits a dual role of both iron reserves and an inflammation indicator. High ferritin levels in patients with GDM can be due to chronic inflammation (IL-6, TNF-α-driven ferritin synthesis) and insulin resistance (enhanced intestinal iron absorption) contributes to paradoxical iron overload (39), which may impair placental function via oxidative stress (ROS) (40) and provide a possible explanation for the negative relationship between ferritin level and neonatal birthweight. The low ferritin levels in low-FT4 individuals and the elevated ferritin levels in those with GDM may have counteracting effects, potentially maintaining neonatal birth weight within the normal range. This phenomenon could mask the metabolic abnormalities in offspring associated with these two maternal conditions.

Furthermore, the study also confirmed a significant positive association between GDM and both HDP and preterm delivery after confounder adjustment. The potential mechanism may involve diabetes-related polyhydramnios, leading to overpressure on the cervix (41) and elevated inferior abdominal pressure.

The identification of trimester-specific thyroid function patterns in patients with GDM highlights the necessity for dynamic monitoring of thyroid hormones during pregnancy, especially in high-risk populations. Additionally, our findings suggest that maternal ferritin levels may act as a mediator in the association between GDM/thyroid dysfunction and neonatal weight, offering a potential biomarker for risk stratification. Clinically, these results advocate for integrated screening protocols that simultaneously evaluate thyroid function and glucose homeostasis in pregnant women, enabling timely interventions to mitigate the risks of preterm birth, HDP, and fetal overgrowth. Future research should explore mechanistic links and intervention strategies to optimize pregnancy management in high-risk populations.

Several limitations should be acknowledged. The retrospective design may introduce selection bias and unaccounted confounders, such as dietary habits and other lifestyle factors. Incomplete follow-up data, particularly for third-trimester thyroid function tests, could compromise the accuracy of late-pregnancy risk assessments. The lack of detailed treatment records for the patients with GDM precludes an analysis of how glycemic control or medication use may modify outcomes. Moreover, the single-center Chinese cohort may limit generalizability to other populations.

## 5 Conclusion

This study demonstrates that trimester-specific maternal thyroid dysfunction differentially influences pregnancy outcomes, with the effects amplified by GDM status and partially mediated by ferritin levels. The relationship between thyroid hormones and glucose metabolism underscores the necessity of comprehensive endocrine evaluations during pregnancy. Our findings advocate for personalized antenatal care that integrates thyroid function monitoring with metabolic and nutritional assessments, particularly in women with GDM or obesity. By addressing these interconnected pathways, clinicians may better predict and prevent complications, ultimately improving maternal and neonatal health outcomes.

## Data Availability

The original contributions presented in the study are included in the article/Supplementary Material. Further inquiries can be directed to the corresponding author.
