# Supplementary material for: Association of maternal thyroid function and gestational diabetes with pregnancy outcomes: a retrospective cohort study
Source: Front Endocrinol (Lausanne). 2025 Jun 6;16:1555409. doi: 10.3389/fendo.2025.1555409 (PMC12178882; doi:10.3389/fendo.2025.1555409)
Supplement: Supplementary file 1 [file Table1.docx]

Supplementary Material

**Table S1. Trimester-specific reference range for FT4 levels.**

|  | **p2.5** | **p5** | **p10** | **p90** | **p95** | **p97.5** |
| --- | --- | --- | --- | --- | --- | --- |
| FT4* (ng/dl) | 13.00 | 13.51 | 14.16 | 19.97 | 21.21 | 22.36 |
| FT4§ | 11.97 | 12.44 | 13.00 | 17.94 | 18.96 | 19.95 |
| FT4† | 10.82 | 11.33 | 11.80 | 16.55 | 17.49 | 18.34 |
| TSH* (mIU/L) | 0.06 | 0.16 | 0.34 | 2.74 | 3.32 | 3.92 |
| TSH§ | 0.11 | 0.25 | 0.44 | 2.88 | 3.40 | 3.91 |
| TSH† | 0.28 | 0.48 | 0.74 | 3.26 | 3.81 | 4.37 |

* First trimester, § second trimester, † third trimester. Abbreviation: FT4, free thyroxine.

**Table S2. Odds ratio (95% CI) for adverse pregnancy outcomes and complications in GDM participants compared to those without GDM.**

| **Pregnancy Outcomes** | **Gestational diabetes** | |
| --- | --- | --- |
|  | **Crude OR (95%CI)** | **Adjusted OR (95%CI)** |
| HDP ^a^ | 1.594 (1.456, 1.747)^***^ | 1.227 (1.116, 1.349)^***^ |
| LGA/Macrosomia ^b^ | 1.223 (1.113, 1.343)^***^ | 0.997 (0.905, 1.098) |
| Preterm Birth ^c^ | 1.131 (1.007, 1.270)^*^ | 1.141 (1.013, 1.286)^*^ |

^a^ Adjusted for maternal age, pre-pregnancy BMI, delivery mode; ^b^ Adjusted for sex of newborn, pre-pregnancy BMI, delivery mode; ^c^ Adjusted for maternal age, sex of newborn, pre-pregnancy BMI, delivery mode. “*” denotes p-value < 0.05, “***” < 0.001. Abbreviation: CI, confidence interval; HDP, hypertensive disorders of pregnancy; LGA, large for the gestational age; OR, odds ratio.
